# Mechanisms of Strigolactone-Regulated Abiotic Stress Responses in Plants

**DOI:** 10.3390/plants14162582

**Published:** 2025-08-20

**Authors:** Jie Dong, Hailin Fu, Zhenyu Wang, Liwei Zhang, Ziyi Liu, Yulin Hu, Fafu Shen, Wei Wang

**Affiliations:** College of Agronomy, Shandong Agricultural University, No. 61 Daizong Street, Tai’an 271018, China; 18853812815@163.com (J.D.); godzzz050102@126.com (H.F.); wzy181901sdau@163.com (Z.W.); 18660839125@163.com (L.Z.); lzy17662693819@163.com (Z.L.); 18132922760@139.com (Y.H.); cotton1@sdau.edu.cn (F.S.)

**Keywords:** strigolactones, abiotic stress, molecular regulation, physiological and biochemical responses, morphological plasticity

## Abstract

Abiotic stresses, such as heat, cold, drought, and salt, pose severe challenges to global agriculture, with climate change exacerbating these threats and intensifying risks to crop productivity and food security. Strigolactones (SLs), a class of phytohormones, play pivotal roles in mediating plant development and enhancing stress resilience. This review highlights the multifaceted mechanisms through which SLs regulate plant responses to abiotic stresses, integrating molecular, physiological, biochemical, and morphological dimensions. Molecularly, SLs regulate the expression of stress-responsive genes, such as those encoding antioxidant enzymes and mitogen-activated protein kinase (MAPK), to enhance plant acclimation and survival under abiotic stress conditions. Moreover, genes involved in SL biosynthesis and signaling pathways are indispensable in these processes. Physiologically and biochemically, SLs improve resilience by modulating photosynthesis, stomatal closure, reactive oxygen species (ROS) metabolism, and osmotic adjustment. Morphologically, SLs modulate leaf morphology, shoot development, and root architecture, enhancing plant stress tolerance. Collectively, SLs emerge as key regulators of plant tolerance to abiotic stresses, offering promising strategies for advancing crop improvement and securing agricultural sustainability in the face of climate change.

## 1. Introduction

Plants, as stationary organisms, must continuously acclimate to fluctuating environmental conditions to survive. Adverse environmental factors, including extreme temperatures, drought, and salinity, often result in abiotic stresses that hinder plant growth and reduce crop productivity, posing significant challenges to sustainable agriculture [1]. Abiotic stress accounts for an estimated 51–82% of global crop yield losses annually [2]. To mitigate these losses, it is essential to understand how plants respond to environmental challenges. Such knowledge can guide the development of stress-resilient crop varieties, ensuring global food security under changing climatic conditions.

To counteract these adversities, plants have evolved sophisticated mechanisms spanning molecular, physiological, biochemical, and morphological levels [3]. Phytohormones act as key regulators in these processes, integrating growth and stress responses through complex signaling pathways [4]. Among these, strigolactones (SLs) have emerged as critical players. Initially identified in the 1960s as root-secreted compounds that induce parasitic *Striga* germination [5], SLs have since been recognized for their diverse roles in plant development and stress responses [6,7,8,9].

Over 30 SLs, classified into canonical and non-canonical types based on their chemical structures, have been identified across plant species [10]. Canonical SLs, such as strigol and orobanchol, contain a core structure consisting of ABC and D rings, differentiated by C-ring stereochemistry. Non-canonical SLs, like carlactone, retain the D-ring but lack the typical tricyclic lactone structure, often featuring an irregular ring [11,12,13]. This structural diversity may contribute to the versatility of SLs in mediating developmental and stress-related processes.

In addition to their role in mediating symbiosis with arbuscular mycorrhizal fungi [14], SLs regulate critical developmental processes, including the modulation of root architecture [15], inhibition of shoot branching [16,17], and promotion of leaf senescence [18]. Notably, increasing evidence highlights their pivotal role in abiotic stress responses, such as heat [19], cold [20], drought [21,22], and salt stress [23,24], where they mediate stress tolerance through intricate mechanisms spanning molecular, physiological, biochemical, and morphological dimensions.

This review aims to elucidate the multifaceted mechanisms by which SLs regulate plant responses to abiotic stresses, emphasizing their roles at molecular, physiological, biochemical, and morphological levels. Understanding these mechanisms offers opportunities to enhance crop resilience and optimize agricultural systems under environmental stress.

## 2. SLs Biosynthesis and Signaling Pathways

**Figure 1:**
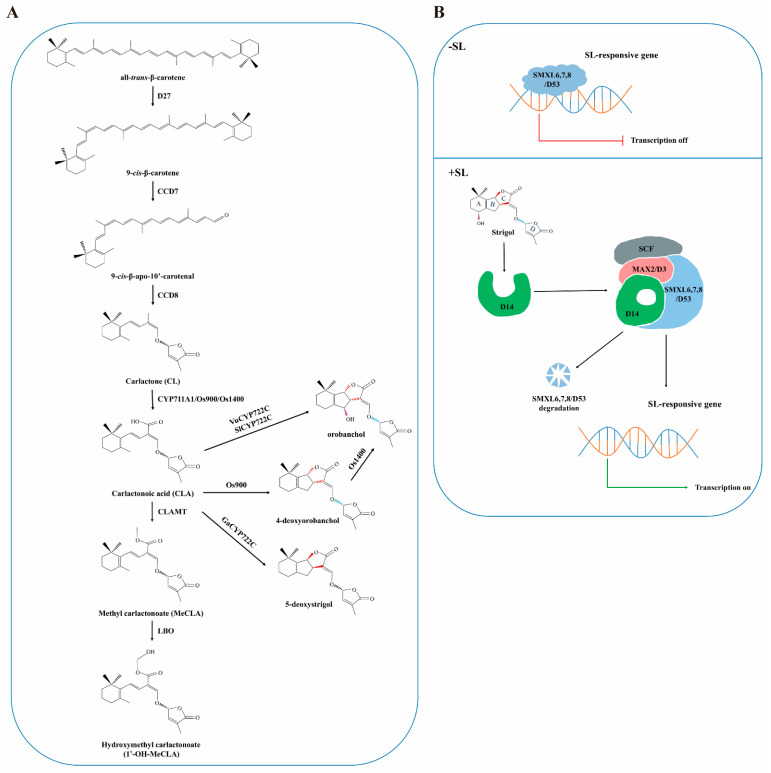
The proposed SL biosynthetic and signaling pathways. (**A**) SL biosynthetic pathway. The biosynthesis of SLs begins with all-*trans*-β-carotene, which is isomerized to 9-*cis*-β-carotene by D27. 9-*cis*-β-carotene is subsequently converted into carlactone (CL) through CCD7 and CCD8. In *Arabidopsis*, CYP711A1 oxidizes CL to form carlactonoic acid (CLA). CLA is further methylated to MeCLA by CLAMT, and then hydroxylated to 1′-OH-MeCLA by LBO. While this pathway represents a general SL biosynthetic mechanism, species-specific variations exist. In rice, Os900 and Os1400 convert CL to CLA. Os900 also catalyzes B-C ring closure, stereo-selectively converting CLA to 4-deoxyorobanchol (4DO), which is hydroxylated by Os1400 to form orobanchol. In cowpea and tomato, CYP722C directly converts CLA into orobanchol, bypassing the 4DO intermediate. In cotton, CYP722C converts CLA to 5-deoxystrigol (5DS). (**B**) SL signaling pathway. In the absence of SLs, transcription of SL-responsive genes is repressed by SL repressors. When SLs are present, they are recognized and bound by the SL receptor D14, which undergoes conformational changes. D14 then interacts with the F-box protein (AtMAX2/OsD3) of the SKP1-CULLIN-F-BOX (SCF) complex and the SL repressors (SUPPRESSORS OF MAX2 1-LIKE6,7,8; AtSMXL6,7,8/OsD53), targeting the repressors for degradation. This degradation releases the repression, enabling the transcription of SL-responsive genes.

The regulation of SL biosynthesis has been extensively investigated, primarily through genetic studies on shoot branching mutants, which have led to the identification of key enzymes involved in this pathway. SL biosynthesis begins with the isomerization of all-*trans*-β-carotene to 9-*cis*-β-carotene, catalyzed by the carotenoid isomerase DWARF27 (D27). This is followed by sequential cleavage reactions mediated by carotenoid cleavage dioxygenases 7 (CCD7) and CCD8 convert 9-*cis*-β-carotene into carlactone (CL) [25]. In *Arabidopsis thaliana* (L.) Heynh. (*Arabidopsis*), the cytochrome P450 monooxygenase MORE AXILLARY GROWTH 1 (MAX1, CYP711A1) oxidizes CL into carlactonoic acid (CLA) [26]. CLA is then methylated into methyl carlactonoate (MeCLA) by CLA METHYLTRANSFERASE (CLAMT) [27], which is subsequently hydroxylated into hydroxymethyl carlactonoate (1′-OH-MeCLA) by LATERAL BRANCHING OXIDOREDUCTASE (LBO), a 2-oxoglutarate and Fe (II)-dependent dioxygenase (Figure 1A) [28].

Interestingly, species-specific variations in SL biosynthesis pathways have been observed. In *Oryza sativa* L. (rice), five homologs of MAX1 have been identified. Among these, Os900 (CYP711A2) and Os1400 (CYP711A3) convert CL into CLA [29]. Os900 further facilitates B-C ring closure, converting CLA into 4-deoxyorobanchol (4DO), a major SL in rice [30]. Os1400 then hydroxylates 4DO to form orobanchol [31]. In other plants, such as *Vigna unguiculata* (L.) Walp. (cowpea) and *Solanum lycopersicum* L. (tomato), the enzyme CYP722C directly converts CLA into orobanchol, bypassing the 4DO intermediate [32]. Similarly, in *Gossypium arboreum* L. (*G. arboreum*), CYP722C converts CLA into 5-deoxystrigol (5DS) (Figure 1A) [33]. The observed species-specific differences in SL biosynthetic pathways suggest a potential evolutionary diversification, which may contribute to ecological adaptation.

Although substantial advances have been made in elucidating the biosynthetic pathways of SLs, their endogenous levels in most plant species remain extremely low. This limitation has prompted the development and application of synthetic SL analogs to facilitate investigations into SL functions. Among these, GR24 is the most widely used synthetic SL analog and serves as a reference compound in SL research [34].

SL signaling pathways have been well-characterized in *Arabidopsis* and rice [35], revealing a conserved mechanism involving receptor activation, repressor degradation, and downstream gene expression (Figure 1B). SL perception is mediated by receptors such as AtD14 and OsD14, members of the α/β hydrolase family. Upon SL binding, the receptor undergoes conformational changes, enabling interaction with F-box proteins (AtMAX2/OsD3) of the SKP1-CULLIN-F-BOX (SCF) complex and repressor proteins (AtSMXL6,7,8/OsD53). The SCF complex ubiquitinates these repressors, targeting them for proteasomal degradation. This derepression promotes the expression of SL-responsive genes, which regulate various developmental and stress-related processes [36,37,38,39,40].

## 3. Role of SLs in Regulating Plant Responses to Heat Stress

At the molecular level, SLs regulate the expression of heat stress-responsive genes to protect plants from heat-induced damage. In tomato, SLs activate the expression of genes encoding antioxidant enzymes (superoxide dismutase [SOD], ascorbate peroxidase [APX], glutathione reductase [GR], monodehydroascorbate reductase [MDAR], and dehydroascorbate reductase [DHAR]) and heat shock proteins (HSPs) [19]. HSPs serve as molecular chaperones, helping maintain protein integrity by assisting in proper folding, preventing denaturation, and preserving overall cellular structure during heat stress [41]. Moreover, key genes involved in SL biosynthesis and signaling pathways, such as *CCD7*, *CCD8*, *MAX1*, and *MAX2*, are upregulated under both heat and cold stress conditions in tomato, enhancing SL-mediated stress tolerance [19].

Physiologically and biochemically, heat stress disrupts photosynthesis, accelerates water loss, induces reactive oxygen species (ROS) accumulation, and compromises membrane stability, all of which impair plant growth and productivity [42]. SLs regulate water-use efficiency and stomatal conductance, reducing excessive water loss under heat stress. For instance, in tomato, SL-deficient mutants exhibit increased stomatal conductance and excessive water loss, whereas GR24 application reduces stomatal conductance, promoting water retention and improving heat tolerance [19]. SLs also enhance antioxidant defenses by boosting the activities of key enzymes, including SOD, APX, GR, MDAR, and DHAR, thereby mitigating oxidative damage and supporting plant survival under high temperatures [19].

Morphologically, SLs mitigate the adverse impacts of heat stress on plant growth by promoting root and leaf development. In cool-season plants, heat stress often inhibits root growth, increasing their vulnerability to elevated temperatures. GR24 application counteracts this inhibition by enhancing crown root elongation in *Festuca arundinacea* (tall fescue). This effect is associated with the upregulation of cell-cycle-related genes, including *Proliferating Cell Nuclear Antigen* (*PCNA*), *Cyclin-D2* (*CycD2*), and *Cyclin-Dependent Kinase B* (*CDKB*), alongside the downregulation of auxin transport-related genes, such as *PIN-FORMED 1* (*PIN1*), *PIN2*, and *PIN5* [43]. Similarly, GR24 treatment enhances leaf elongation under heat stress, paralleling its effects on root elongation in tall fescue [44].

## 4. Role of SLs in Regulating Plant Responses to Cold Stress

At the molecular level, SLs regulate the expression of cold-responsive genes to mitigate cold-induced damage. In *Brassica rapa* L. (*B. rapa*), GR24 induces the expression of antioxidant enzyme genes (*CAT*, *SOD*, *APX*, *POD*), nicotinamide adenine dinucleotide phosphate (NADPH) oxidase genes (*RbohA*-*D*, *RbohF*-*G*), mitogen-activated protein kinase (MAPK) genes (*MPK3*, *MPK6*), and cold-related genes (*COR*, *ICE1*), thereby improving cold tolerance [20]. Genes involved in SL biosynthesis and signaling pathways are also crucial for plant responses to cold stress. SL-deficient mutants of *Arabidopsis* and *Pisum sativum* L. (pea) exhibit significant reductions in photosynthetic capacity under cold stress, underscoring the importance of SLs in maintaining photosynthetic activity and cold tolerance [45]. *Arabidopsis* SL receptor *d14* mutants display reduced freezing tolerance, with lower survival rates and higher ion leakage under cold conditions [46]. In *Arabidopsis*, the transcription factor WRKY41 represses the expression of cold-responsive gene *DEHYDRATION RESPONSE ELEMENT BINDING FACTOR 1* (*DREB1*) by binding to its promoter, thereby impairing cold tolerance. SLs, through the F-box protein MAX2 of the SCF complex, promote the degradation of WRKY41, thereby alleviating this repression and allowing *DREB1* upregulation, which is essential for cold stress tolerance. Furthermore, SL-mediated degradation of SMXL proteins enhances anthocyanin biosynthesis, contributing to cold tolerance in *Arabidopsis* [46].

Physiologically and biochemically, cold stress adversely affects plant growth by inhibiting photosynthesis and transpiration, reducing water and nutrient uptake, and inducing oxidative damage [47]. GR24 mitigates these effects by preserving PSII quantum efficiency and reducing the accumulation of ROS such as hydrogen peroxide (H_2_O_2_) and superoxide anions (O_2_^•−^) in *Vigna radiata* (L.) R. Wilczek (mung bean) seedlings [48]. GR24 also enhances proline and soluble sugar levels, improving water retention and alleviating dehydration caused by chilling stress [48]. In *B. rapa*, GR24 significantly improves photosynthetic efficiency, antioxidant enzyme activities, and proline and soluble protein contents, while reducing ROS levels and relative conductivity, thereby enhancing cold tolerance [20]. Similarly, in tomato, GR24 enhances the activities of antioxidant enzymes, including SOD, APX, GR, MDAR, and DHAR, all of which help plants alleviate oxidative stress induced by cold conditions [19].

Morphologically, SLs modulate leaf development under cold stress. In pea, SL-deficient and SL-response mutants (*rms5-3*, *rms3-1*, *rms4-1*) develop more leaves than wild-type (WT) plants after dark chilling treatments, emphasizing the importance of SLs in regulating leaf development under cold conditions [45]. Similarly, in *Arabidopsis*, dark chilling significantly reduces rosette areas in *max4-1* and *max2-1* mutants relative to WT, further underscoring SLs’ role in leaf development during cold stress [45].

## 5. Role of SLs in Regulating Plant Responses to Drought Stress

At the molecular level, GR24 enhances the expression of genes related to photosynthesis (*PpPEPCK*, *PpRuBPC*, *PpPGK*, *PpGAPDH*, *PpFBA*, and *PpSBPase*) and root development (*PpACAT*, *PpMFP2*, *PpAGT2*, *PpIVD*, *PpMCCA*, and *PpMCCB*) under drought stress in *Pennisetum purpureum* Schum. (*P. purpureum*), contributing to improved drought resilience [22]. SLs and ABA share a carotenoid-derived biosynthetic pathway, and their interaction modulates drought responses. For example, the *Arabidopsis* SL receptor *d14* mutant exhibits hypersensitivity to drought stress, characterized by slower abscisic acid (ABA)-induced stomatal closure and reduced anthocyanin accumulation [49]. Similarly, the *Hordeum vulgare* L. (barley) SL receptor *hvd14.d* mutant displays increased drought sensitivity, likely due to disrupted ABA metabolism or signaling, despite unchanged ABA levels [50]. Overexpression of the SL receptor *D14* gene from *Zea mays* L. (maize) in *Arabidopsis* enhances drought tolerance [51]. In *Arabidopsis*, SL-signaling mutants such as *max2* demonstrate increased drought sensitivity due to impaired stomatal closure and excessive water loss. Unlike *max3* and *max4*, the drought-sensitive phenotype of *max2* could not be rescued by GR24 treatment [21]. Similarly, overexpression of *Glycine max* (L.) Merr. (soybean) SL-signaling gene *GmMAX2a* in *Arabidopsis* enhances drought tolerance [52]. Moreover, the *Arabidopsis smxl6,7,8* triple mutant exhibits improved drought tolerance compared to WT, with elevated anthocyanin biosynthesis and increased sensitivity to ABA [53].

Physiologically and biochemically, SLs contribute to efficient water retention by modulating stomatal behavior under drought conditions. GR24 reduces stomatal aperture and limits transpiration-driven water loss in crops such as *Vitis vinifera* L. (grape), *Triticum aestivum* L. (wheat), and maize [54,55,56,57]. This regulation is closely linked to ABA signaling. In *Arabidopsis*, SL-deficient mutants (*max3* and *max4*) exhibit impaired ABA-mediated stomatal closure, resulting in excessive water loss under drought stress [21]. GR24 application restores the WT phenotype in these mutants, underscoring the synergistic interaction between SLs and ABA [21]. Similarly, SL-deficient mutants in *Lotus japonicus* (*Ljccd7*-silenced) and tomato (*Slccd7*-silenced) display reduced sensitivity to ABA-mediated stomatal closure, further highlighting the role of SLs in enhancing ABA signaling [58,59].

Drought stress significantly limits photosynthesis by reducing CO_2_ intake through stomatal closure and disrupting chloroplast structure [60,61]. SLs mitigate these effects by preserving chlorophyll levels and maintaining photosynthetic efficiency. GR24 alleviates drought-induced reductions in chlorophyll content in crops such as grape, wheat, maize, *P. purpureum*, *Malus hupehensis* Rehd. (crab apple), and *B. rapa* [54,55,56,57,62,63,64], allowing plants to sustain higher photosynthetic rates under drought stress.

ROS accumulation, including H_2_O_2_ and O_2_^•−^, causes oxidative damage to cellular components under drought conditions [65]. SLs alleviate oxidative stress by enhancing antioxidant enzyme activities, such as SOD, POD, CAT, and APX. GR24 application boosts these enzyme activities in crops like grape, wheat, maize, alfalfa, crab apple, and *B. rapa*, thereby reducing ROS levels and protecting cells from oxidative damage [54,55,56,57,62,64,66].

Osmotic adjustment is another critical strategy for drought tolerance, with plants accumulating osmolytes such as proline and soluble sugars to maintain turgor pressure and protect cellular integrity under dehydration [67]. GR24 promotes osmolyte accumulation in wheat, alfalfa, and maize, enhancing drought tolerance [57,66,68]. Additionally, GR24 stabilizes cell membranes by reducing lipid peroxidation and electrolyte leakage, as indicated by lower malondialdehyde (MDA) levels and electrolyte leakage in crops like wheat, grape, *Dracocephalum kotschyi Boiss* (*D. kotschyi*), and crab apple [54,62,64,69,70].

Morphologically, SLs regulate root architecture to improve water uptake under drought conditions. GR24 promotes root growth, increases water absorption, and enhances drought resistance across various crops. For example, GR24 promotes root activity and drought resistance in crab apple [62], while significantly increasing root length in *Medicago Sativa* L. (alfalfa) and *P. purpureum* under drought stress [63,66]. Additionally, in wheat, GR24 enhances the root-to-shoot ratio, optimizing water-use efficiency under drought conditions [55].

## 6. Role of SLs in Regulating Plant Responses to Salt Stress

At the molecular level, SLs regulate key genes involved in stress responses. In *Brassica napus* L. (*B. napus*), GR24 modulates the expression of genes related to photosynthesis, tryptophan metabolism, and phytohormone signaling pathways under salt stress [71]. In *Cucumis sativus* L. (cucumber), GR24 enhances the expression of genes encoding antioxidant enzymes, NADPH oxidase, calcium-dependent protein kinases (CDPKs), salt overly sensitive 1 (*SOS1*), CBL-interacting protein kinase 2 (*CIPK2*), and calcineurin B-like protein 3 (*CBL3*), thereby alleviating salt-induced damage [72]. Transcriptomic analyses further reveal that GR24 influences pathways associated with H_2_O_2_ and MAPK signaling cascades, which play crucial roles in salt stress alleviation in cucumber [73].

Genes involved in SL biosynthesis and signaling pathways are crucial for salt stress tolerance. Salt stress induces the expression of SL biosynthesis genes, such as *CCD7* and *CCD8*, and signaling components like *MAX2*. In *Sesbania cannabina* (Retz.) Pers. (*S. cannabina*), salt stress and exogenous applications of ABA and H_2_O_2_ upregulate the expression of *CCD7*, *CCD8* (in roots), and *MAX2* (in shoots), promoting SL biosynthesis and enhancing salt tolerance [23]. Conversely, inhibiting SL biosynthesis or scavenging H_2_O_2_ suppresses SL production, highlighting the importance of ROS and ABA in SL biosynthesis under salt stress [23]. Moreover, SL signaling mutants such as *max2* in *Arabidopsis* exhibit increased sensitivity to salt stress, underscoring the importance of *MAX2* in SL-mediated salt stress responses [21].

Physiologically and biochemically, salt stress impairs photosynthesis due to stomatal closure and reduced levels of photosynthetic pigments [74]. SLs mitigate these effects by enhancing photosynthetic efficiency. In crops like *B. napus*, rice, *Salvia nemorosa* L. (*Salvia*), tomato, and maize, GR24 application increases stomatal conductance, chlorophyll content, and net photosynthetic rate, allowing plants to maintain higher photosynthetic capacity under salt stress [71,75,76,77,78,79].

Salt stress triggers ROS accumulation, leading to oxidative damage to cellular components such as DNA, proteins, and lipids [80]. SLs alleviate oxidative stress by upregulating antioxidant enzyme activities. GR24 treatment enhances the activities of enzymes such as POD, SOD, CAT, APX, and GR in salt-stressed plants, including *B. napus*, rice, *Salvia*, *Helianthus annuus* L. (sunflower), tomato, cucumber, and wheat. This results in reduced levels of H_2_O_2_ and MDA, thereby minimizing oxidative damage [24,71,72,75,76,77,78,81,82].

Salt stress disrupts ionic balance by causing excessive sodium ion (Na^+^) accumulation, which interferes with cellular functions [83]. SLs restore ionic homeostasis by increasing potassium (K^+^) and calcium (Ca^2+^) levels while reducing Na^+^ accumulation. For example, GR24 application increases K^+^ and Ca^2+^ levels in sunflower and maize while reducing Na^+^ accumulation, thus enhancing salt tolerance [79,81]. Similarly, in bread wheat, GR24 maintains higher K^+^ levels and lower Na^+^ accumulation under saline conditions [82]. Additionally, SLs promote osmotic adjustment by enhancing osmoprotectant accumulation. GR24 treatment increases proline content in *Salvia*, sunflower, cucumber, tomato, and wheat under salt stress, helping plants maintain osmotic balance [72,76,78,81,82].

Morphologically, SLs improve root and shoot development, which are essential for plant responses to saline environments. GR24 application promotes root and shoot growth in *B. napus* and sunflower under salt stress [71,84,85]. In rice, where salt stress significantly reduces growth traits such as root length and plant height, GR24 restores these traits and enhances seedling vigor under saline conditions [75]. Similarly, GR24 treatment improves root and shoot length in tomato, demonstrating its effectiveness in counteracting salt-induced growth inhibition [76,77].

**Figure 2:**
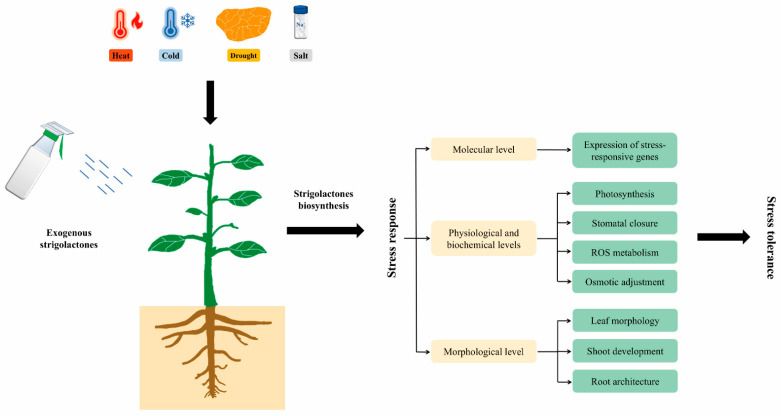
The proposed model for the effects of SLs on plant responses to abiotic stresses (heat, cold, drought, and salt stresses) at multiple levels. At the molecular level, SLs enhance plant resistance to abiotic stresses by regulating the expression of stress-responsive genes. At the physiological and biochemical levels, SLs improve plant resistance to abiotic stresses by modulating photosynthesis, stomatal closure, ROS metabolism, and osmotic adjustment. At the morphological level, SLs enhance plant resistance to abiotic stresses by regulating leaf morphology, shoot development and root architecture. This integrative model highlights SLs’ critical role in improving plant stress tolerance.

In summary, SLs regulate plant responses to abiotic stresses through integrative mechanisms at the molecular, physiological, biochemical, and morphological levels, which are illustrated in Figure 2. Table 1 summarizes the effects of exogenous GR24 application under abiotic stress conditions, while Table 2 highlights the roles of genes involved in SL biosynthesis and signaling pathways in abiotic stress tolerance.

## 7. Conclusions and Future Perspectives

SLs have emerged as pivotal regulators of plant responses to a variety of abiotic stresses, including heat, cold, drought, and salt. At the molecular level, SLs modulate the expression of stress-responsive genes, thereby enhancing plant resilience. Moreover, genes involved in SL biosynthesis and signaling pathways are fundamental to the regulation of abiotic stress responses. Physiologically and biochemically, SLs enhance tolerance by preserving photosynthetic efficiency, modulating stomatal behavior to reduce water loss, regulating ROS metabolism to alleviate oxidative damage, and facilitating osmotic adjustment. At the morphological level, SLs influence leaf morphology, shoot development, and root architecture, thereby optimizing plant structure for stress resilience.

Some reviews have elaborated on the hormonal crosstalk between SLs and other phytohormones, including ABA, auxin, cytokinin (CK), ethylene (ET), salicylic acid (SA), and jasmonic acid (JA) [87,88,89,90]. For instance, SLs have been reported to enhance ABA-dependent heat and cold tolerance in tomato [19], and interact synergistically with ABA, SA, and JA to improve drought responses [50,68,69,70]. While these interactions highlight the integrative role of SLs within the broader hormonal network, the underlying molecular mechanisms remain insufficiently defined. Future research should focus on deciphering shared signaling components, transcriptional regulators, and downstream effectors that mediate SL-hormone cross-regulation under abiotic stress.

To accelerate SL-based crop improvement, several biotechnological strategies have been employed. Genetic disruption or silencing of *CCD7*, *CCD8*, *MAX1*, and *MAX2* by CRISPR/Cas9 or virus-induced gene silencing (VIGS) has been shown to reduce heat and cold tolerance in tomato, underscoring their importance in abiotic stress adaptation [19]. Conversely, heterologous expression of the maize SL receptor gene *D14* in *Arabidopsis* significantly improved drought tolerance [51], and similar enhancement was observed when the soybean SL signaling gene *GmMAX2a* was overexpressed in *Arabidopsis* [52]. In addition, combined transcriptomic and metabolomic analyses have uncovered the mechanisms by which exogenous SLs modulate drought responses in elephant grass [22]. These studies collectively demonstrate the potential of genetic engineering and molecular breeding to manipulate SL pathways for improved stress resilience.

Although SL functions have been partially characterized in major crops such as wheat and maize, the detailed molecular regulatory pathways, particularly those under field-relevant stress conditions, remain largely unexplored. Translating mechanistic insights from model plants to diverse agricultural species under field conditions remains a pressing challenge. Large-scale genome-wide association studies (GWAS), CRISPR-based functional genomics, and field-level phenotyping will be crucial for dissecting SL-regulated networks in crop species and harnessing them for future breeding programs.

To fully elucidate the complexity of SL-mediated stress regulation, integrative multi-omics approaches are indispensable. The convergence of transcriptomics, proteomics, metabolomics, phenomics, and epigenomics will offer a systems-level perspective of how SLs coordinate developmental and environmental response networks. Advanced tools such as spatial transcriptomics and single-cell RNA sequencing also hold great promise for unraveling tissue- and cell-type-specific dynamics of SL biosynthesis and signaling under abiotic stress.

SLs and their synthetic analogs possess diverse functions, influencing crop yield and quality, stress tolerance, and overall agricultural sustainability [9,91]. Beyond the widely used GR24, analogs such as Nijmegen-1 and methyl phenlactonoates (MPs) have shown strong bioactivity, particularly in triggering suicidal germination of parasitic weeds [91,92,93], contributing to environmentally friendly weed control strategies. AB01, another synthetic SL analog, has been reported to significantly reduce *Striga* biomass while improving drought and salinity tolerance in crops [9,91]. However, despite their promising potential, several challenges hinder the widespread agricultural application of these analogs, including chemical instability, environmental safety concerns, regulatory constraints, and the need for cost-effective synthesis methods. Addressing these limitations through structural optimization and policy support will be key to their future agronomic use.

In conclusion, SLs represent a promising target for improving crop resilience to abiotic stress and mitigating the impacts of climate change on agriculture. A deeper understanding of SL-centered signaling networks will facilitate the development of innovative and sustainable strategies to enhance crop performance, contributing to global food security under increasingly challenging environmental conditions.

## Figures and Tables

**Table 1:** The effects of exogenous GR24 application on plant responses to heat, cold, drought, and salt stress.

Stress	Species	GR24 Treatment	Effect	Reference
Heat	*Festuca arundinacea*	0.01 μM	Increases: crown root elongation; cell cycle-related genes expression Decreases: auxin transport related genes expression	[43]
		0.01 μM	Increases: leaf elongation; cell division; cell cycle-related genes expression Decreases: auxin transport related genes expression	[44]
Cold	*Brassica rapa*	0.1 μmol L^−1^	Increases: SOD, POD, CAT and APX activities; soluble protein and proline contents; *SOD*, *POD*, *CAT*, *APX*, *MPK3*, *MPK6*, *ICE1* and *COR* expression Decreases: MDA and H_2_O_2_ contents	[20]
	*Vigna radiata*	1 and 10 μM	Increases: RWC; SOD, PAL, TAL and LOX activities; total soluble sugar and proline contents Decreases: O_2_^•−^, H_2_O_2_, phenolics and MDA contents	[48]
Heat and Cold	*Solanum lycopersicum*	3 µM	Increases: SOD, APX, GR, MDAR and DHAR activities; leaf ABA content; *NCED6*, *HSP70* and *CBF1* expression	[19]
Drought	*Brassica rapa*	10 μM	Increases: photosynthesis traits; antioxidant defenses Decreases: MDA content	[64]
	*Dracocephalum kotschyi*	10 µM	Increases: fresh and dry weights; essential oil content and yield Decreases: electrolyte leakage; MDA and H_2_O_2_ contents	[70]
	*Malus hupehensis*	1 µM	Increases: chlorophyll contents; photosynthetic parameters; antioxidant capacity Decreases: O_2_^•−^, H_2_O_2_ and MDA contents	[62]
	*Medicago sativa*	0.1 µM	Increases: root, stem and leaf FW; plant height; root length; POD and CAT activities in leaves; POD, CAT and SOD activities in roots; leaves and roots soluble protein contents	[66]
	*Pennisetum purpureum*	1, 3, 5 and 7 μmol L^−1^	Increases: root development; water-use efficiency; photosynthesis; photosynthetic enzyme activity	[63]
		3 μmol L^−1^	Increases: *PpAGT2*, *PpIVD*, *PpMCCA* and *PpMCCB* expression to control root development; *PpPEPCK*, *PpRuBPC*, *PpPGK*, *PpGAPDH*, *PpFBA* and *PpSBPase* expression to regulate photosynthetic capacity Decreases: *PpACAT* and *PpMFP2* expression to regulate root development	[22]
	*Triticum aestivum*	10 μM	Increases: RWC; membrane stability index; POD, CAT and APX activities Decreases: electrolyte leakage; MDA content	[69]
		10 μM	Increases: stomatal conductance; photosynthetic rate; proline and soluble sugar contents Decreases: H_2_O_2_ content	[68]
		5 and 10 µM	Increases: root and shoot DW; transpiration rate and stomatal conductance; photosynthetic rate; SOD, POD, CAT and APX activities Decreases: H_2_O_2_ content	[55]
	*Vitis vinifera*	1, 3 and 5 μM	Increases: RWC; chlorophyll content and photosynthetic rate; antioxidant capacity Decreases: stomatal opening; electrolyte leakage; H_2_O_2_ and MDA contents	[54]
	*Zea mays*	10 and 20 µM	Increases: water relations; gas exchange parameters; photosynthetic pigments; antioxidant enzymes activities	[56]
		0.001, 0.01 and 0.1 mg L^−1^	Increases: stomatal conductance; water use efficiency; gas exchange characteristics; net CO_2_ assimilation rate; chlorophyll content; antioxidant enzymes activities; leaf ascorbic acid; total phenolics; glycine betaine; free proline	[57]
Salt	*Brassica napus*	0.18 μM	Increases: root and shoot FW and DW; transpiration rate and stomatal conductance; chlorophyll content and photosynthetic rate; SOD and POD activities Decreases: MDA content	[71]
	*Cucumis sativus*	1.0 μmol L^−1^	Increases: SOD, POD, CAT and APX activities; proline content; *SOD*, *POD*, *CAT* and *APX* expression Decreases: electrolyte leakage; O_2_^•−^ and H_2_O_2_ contents	[72]
		10 μM	Increases: SOD, POD, CAT and APX activities; AsA and GSH contents Decreases: MDA, H_2_O_2_ and O_2_^•−^ contents; proline content	[24]
	*Helianthus annuus*	3.35, 33.5 and 335 nM	Increases: root and shoot FW and DW; gas exchange attributes; osmotic potential and RWC	[84]
		0.001, 0.01 and 0.1 mg L^−1^	Increases: plant biomass and shoot length; carotenoids contents; shoots and roots Na^+^, K^+^ and Ca^2+^ contents	[85]
		0.001, 0.01 and 0.1 mg L^−1^	Increases: callus DW and FW; SOD, POD and CAT activities; free protein, free proline, glycine betaine, K^+^ and Ca^2+^ contents Decreases: MDA and H_2_O_2_ contents; Na^+^ content	[81]
	*Oryza sativa*	0.1, 0.2, 1 and 5 μM	Increases: root and shoot DW; plant height and root length; transpiration rate and stomatal conductance; chlorophyll content and photosynthetic rate; POD and SOD activities Decreases: MDA content	[75]
	*Salvia nemorosa*	0.1, 0.2, 0.3 and 0.4 μM	Increases: plant growth rate; stomatal conductance; chlorophyll content and photosynthetic rate; SOD, POD, CAT and GR activities; proline and total phenolics contents; essential oil yield and content Decreases: electrolyte leakage, MDA and H_2_O_2_ contents	[78]
	*Solanum lycopersicum*	15 μM	Increases: leaf area; root length; chlorophyll and carotenoid contents; SOD, POD, CAT, APX and GR activities	[77]
		2 µM	Increases: photosynthetic efficiency; antioxidant capacity; proline and protein contents	[76]
	*Triticum aestivum*	0.001, 0.01 and 0.1 mg L^−1^	Increases: net CO_2_ assimilation rate	[86]
		10 µM	Increases: grain yield; proline and glycine betaine contents; APX and POX activities; K^+^/Na^+^ ratio Decreases: electrolyte leakage; H_2_O_2_ and MDA contents	[82]
	*Zea mays*	0.001, 0.01 and 0.1 mg L^−1^	Increases: grains/cob number and main cob diameter; transpiration and stomatal conductance; chlorophyll and carotenoids contents, photosynthetic rate; K^+^ and Ca^2+^ contents Decreases: Na^+^ content	[79]

The molecular weight of GR24 is 298.29. For drought stress, in *Zea mays* (Ref. [57]), 0.01 mg L^−1^ was reported as the optimal concentration. For salt stress, in *Helianthus annuus*, 33.5 nM (Ref. [84]) and 0.01 mg L^−1^ (Refs. [81,85]) were identified as optimal. In *Oryza sativa* (Ref. [75]), the optimal concentration was 1 μM; and in *Zea mays* (Ref. [79]), the optimal concentration was 0.01 mg L^−1^.

**Table 2:** The role of genes involved in SL biosynthesis and signaling pathways in plant responses to heat, cold, drought, and salt stress.

Stress	Species	Gene	Effect	Reference
Cold	*Arabidopsis thaliana*	*MAX3*, *MAX4* and *MAX2*	positively regulate dark chilling tolerance	[45]
		*MAX3*, *MAX4*, *MAX1*, *MAX2*, *D14*, and *SMXL6,7,8*	*MAX1-4* and *D14* positively regulate cold tolerance; *SMXL6,7,8* negatively regulate cold tolerance	[46]
	*Pisum sativum*	*RMS5*, *RMS4* and *RMS3*	positively regulate dark chilling tolerance	[45]
Heat and Cold	*Solanum lycopersicum*	*CCD7*, *CCD8*, *MAX1*, and *MAX2*	positively regulate heat and cold tolerance	[19]
Drought	*Arabidopsis thaliana*	*D14*	positively regulate drought tolerance	[49]
		*SMXL6,7,8*	negatively regulate drought tolerance	[53]
	*Glycine max*	*MAX2a*	positively regulate drought tolerance	[52]
	*Hordeum vulgare*	*D14*	positively regulate drought tolerance	[50]
	*Lotus japonicus*	*CCD7*	positively regulate drought tolerance	[58]
	*Solanum lycopersicum*	*CCD7*	positively regulate drought tolerance	[59]
	*Zea mays*	*D14*	positively regulate drought tolerance	[51]
Drought and Salt	*Arabidopsis thaliana*	*MAX3*, *MAX4* and *MAX2*	positively regulate drought and salt tolerance	[21]

## Data Availability

All data are included in this article.
